# Understanding the Complexities of Functional Ability in Alzheimer’s Disease: More Than Just Basic and Instrumental Factors

**DOI:** 10.2174/1567205011666140317101419

**Published:** 2014-05

**Authors:** Kristin Kahle-Wrobleski, Nicola Coley, Benoit Lepage, Christelle Cantet, Bruno Vellas, Sandrine Andrieu, for the PLASA/DSA Group

**Affiliations:** 1 Lilly Research Laboratories, Eli Lilly and Company, Indianapolis, IN, USA;; 2 Inserm U1027, F-31073, Toulouse, France;; 3 University Toulouse III, Toulouse, F-31073, France;; 4 CHU Toulouse, Department of Epidemiology and Public Health, Toulouse, F-31073, France;; 5 Gerontopole, CHU Toulouse, Department of Geriatric Medicine, F-31059, France, †Members are listed at the end of the manuscript.; † Members are listed at the end of the manuscript.

**Keywords:** Activities of daily living, ADCS-ADL, Alzheimer’s, factor analysis, function, instrumental ADLs.

## Abstract

*
Background*: Dementia of the Alzheimer’s type (AD) is defined by both cognitive and functional decline; new criteria allow for identification of milder, non-functionally impaired patients. Understanding loss of autonomy in AD is essential, as later stages represent a significant burden and cost to patients, their families, and society. The purpose of the present analyses was to determine the factor structure of the Alzheimer’s Disease Cooperative Study–Activities of Daily Living Scale (ADCS-ADL) in a cohort of AD patients. *Methods*: Baseline ADCS-ADL assessments of 734 AD patients from the PLASA study were included in an exploratory factor analysis (EFA). Because the ADCS-ADL was designed to assess change over time, change from baseline scores over 2 years were also analyzed using an EFA. Factorial solutions were evaluated based on cross-loading, non-loadings, and number of items per factor. *Results*: Mean age at baseline was 79.3, mean MMSE was 19.8 and 73.3‰ of participants were female. Baseline data suggested a 4-factor solution that included factors for basic ADLs (BADLs), domestic/household activities, communication/engagement with the environment, and outside activities. The change scores EFA suggested a 2-factor solution of BADLs and instrumental ADLs (IADLs). *Conclusions*: Distinct factors of IADLs should be considered for further validation as areas of attention to catch early functional decline.

## INTRODUCTION

Criteria currently used in clinic and research for identifying dementia due to Alzheimer’s Disease (AD) require both cognitive and functional loss for a diagnosis of dementia [1, 2]. Whereas specific domains of cognition are identified in the diagnostic criteria (e.g., memory, aphasia), functional loss is defined with less granularity. Accurately measuring functional loss is important because the nature and extent of functional losses associated with disease progression help determine the type and level of care needed, ranging from medication management in early stages to full time care or institutionalization in later stages of AD [3]. Functional loss is also a driver of higher costs in more severe stages of dementia [4]. Furthermore, proposed revisions to the current diagnostic criteria allow for a diagnosis of Alzheimer’s disease in milder cognitive stages without significant functional loss [5, 6]. Therefore, it is of crucial importance to understand when and what types of functional losses are likely to occur to help with treatment and care planning even at the earliest stages of the disease, as they may predict later decline [7].

Functional ability as assessed in any disease state, including AD, is typically described in terms of activities of daily living (ADLs) that are divided into broad conceptual categories of basic ADLs (BADLs) and instrumental ADLs (IADLs). Measuring BADLs is straightforward, as basic self-care tasks are generally recognizable in all cultures and include feeding, mobility, toileting, bathing, grooming, and dressing [8, 9]. The measure and conceptualization of IADLs is more complex due to the influence of cultural norms and gender roles that may impact which tasks are even attempted by a patient. As such, scales that measure IADLs tend to include a broad range of activities. A number of scales are used to measure IADLs and ADLs in observational studies and randomized clinical trials of patients with AD. One in particular, the Alzheimer’s Disease Cooperative Study – Activities of Daily Living Scale (ADCS-ADL) [10], was constructed specifically for use with AD patients. The ADCS-ADL is a 23 item scale that includes 6 BADL items and 17 IADL items that provide a total score from 0-78, with a lower score indicating greater severity. Caregivers are asked to rate the degree to which their family member or loved one can perform a variety of tasks.

Given the broad range of IADLs sampled in the ADCS-ADL, different types of IADLs may be grouped together to provide clinicians and family members with more pragmatic indices of function. For example, data from a 6 month observational study of AD patients suggested two IADL factors that the authors called “Domestic activities” and “Communication activities” [11]. Alternatively, a factor-analysis of the 19-item ADCS-ADL scale from a randomized clinical trial of moderate to severe AD patients revealed a four factor structure described as ADL (basic), Higher-level functions, Simple motor skills, and Connectedness/Autonomy [12]. Comparisons between the observational study data and clinical trial data are somewhat limited as the observational study included a range of patients at all severity levels and few exclusion criteria, whereas the clinical trial data included only medically stable and ambulatory patients. Further, both studies included a relatively brief 6 month follow-up period.

Given the varying factor structures previously reported for the ADCS-ADL, the present set of analyses looked at the factor structure of the ADCS-ADL in a cohort of Alzheimer’s disease patients from a longitudinal study to determine the factor structure at baseline. This will provide descriptive categories of ADLs to help conceptualize functional ability in a manner that facilitates communication with family members, patients, and other care partners. Because the ADCS-ADL was designed to assess change over time, the present study also looked at the underlying factors of change scores for scale items using baseline to 2-year endpoint change score. The follow-up period was therefore comparable to the typical 18-24 month follow-up periods in current AD trials. The factor structure at baseline and factor structure of change were compared.

## MATERIAL AND METHODS

Patient data were drawn from the PLASA study, a prospective study of 1131 patients diagnosed with AD according to NINCDS-ADRDA criteria and recruited in France that compared two treatment arms: one receiving usual care and one receiving a comprehensive Alzheimer’s disease management plan. The study methodology was previously described [13]. The intervention had no significant effect on any of the primary or secondary efficacy measures. It was therefore decided that patients from both randomization groups were eligible for the present study. For the baseline analysis, 734 patients with no missing ADCS-ADL items at baseline were included. For the change from baseline calculations, 183 participants had no missing ADCS-ADL items after 2 year follow-up and were included in the analysis. Patients with any missing items on the ADCS-ADL were excluded from the present analysis, as this was meant to be an examination of the scale’s properties rather than an examination of clinical questions. Therefore, no imputation scheme was used for these analyses. Fig. (**1**) shows the flow of patients through the study and reasons for discontinuation. Patients from the PLASA cohort who were not included in this analysis at baseline were significantly older (mean age 80.31 vs. 79.30, p<0.05), had onset of symptoms for a significantly longer period of time (3.76 years vs. 3.55 years, p<0.05), and had significantly more comorbidities (2.16 vs. 2.00, p=0.05). Patients who were excluded from the follow-up analysis due to having missing items on the ADCS-ADL were significantly more functionally impaired at baseline than those included in the follow-up analyses (ADCS-ADL Visit 1 Total=53.15 vs. 58.39, p<0.0001).

An exploratory common factor analysis (EFA) was conducted. Because prior factor analyses of this scale either separated out the BADL items a priori or included a different version of the scale, this approach was viewed as preferable to forcing a factor structure and estimating model fit via a confirmatory factor analysis (CFA). For the purposes of these analyses, questions 6a and 6b (dressing), 16a and 16b (shopping) were treated as separate items because they assess different skills (e.g., being able to choose an outfit vs. being able to perform the mechanics of dressing). This resulted in 25 separate items. Change scores for the second analyses were computed by subtracting the item score at endpoint from the item score at baseline.

A promax rotation was used because the latent factors were correlated with each other as shown by the matrix of inter-factor correlations (See Table **1**). To avoid the potential difficulty with interpretation of oblique rotation results, the factor pattern matrix was used instead of factor loadings to guide interpretation [14]. Criteria to determine how many factors to retain included the Scree Test [15]. The mean eigenvalue rule (retaining factors with eigenvalues greater than the mean eigenvalue) was also considered since using the eigenvalue>1 rule may underestimate the number of factors [16].

To facilitate interpretability, the preferred factor structure was chosen based on minimizing the number of items that cross-loaded on to multiple factors and minimizing the number of non-loading factors. Another consideration was to retain solutions that yielded factors with at least 3 items loading on to that factor [14]. A cut-off of >.30 was used to determine onto which factor an item loaded [17].

Analyses were run first using baseline data. A second set of analyses was run to look at the factor structure of the change score from baseline to endpoint. 

## RESULTS

Baseline descriptive statistics on patient demographics are provided in (Table **2**). Participants were predominantly female and a majority had no more than a high school level of education.

The evolution of disease severity over 2 years, in terms of cognition and function, for the subjects included in the follow-up analysis is shown in (Table **3**). Mean MMSE score declined by approximately 2.6 points, while mean total ADCS-ADL score declined by approximately 9 points, essentially due to decline in IADL items. 

For the baseline analyses, total eigenvalues of the reduced correlation matrix was 9.38, and the average eigenvalue was 0.38. The scree plot of the CFA suggested a 4 factor solution, and this was confirmed by 4 factors having eigenvalues of >0.38. 

**Fig. (2):**
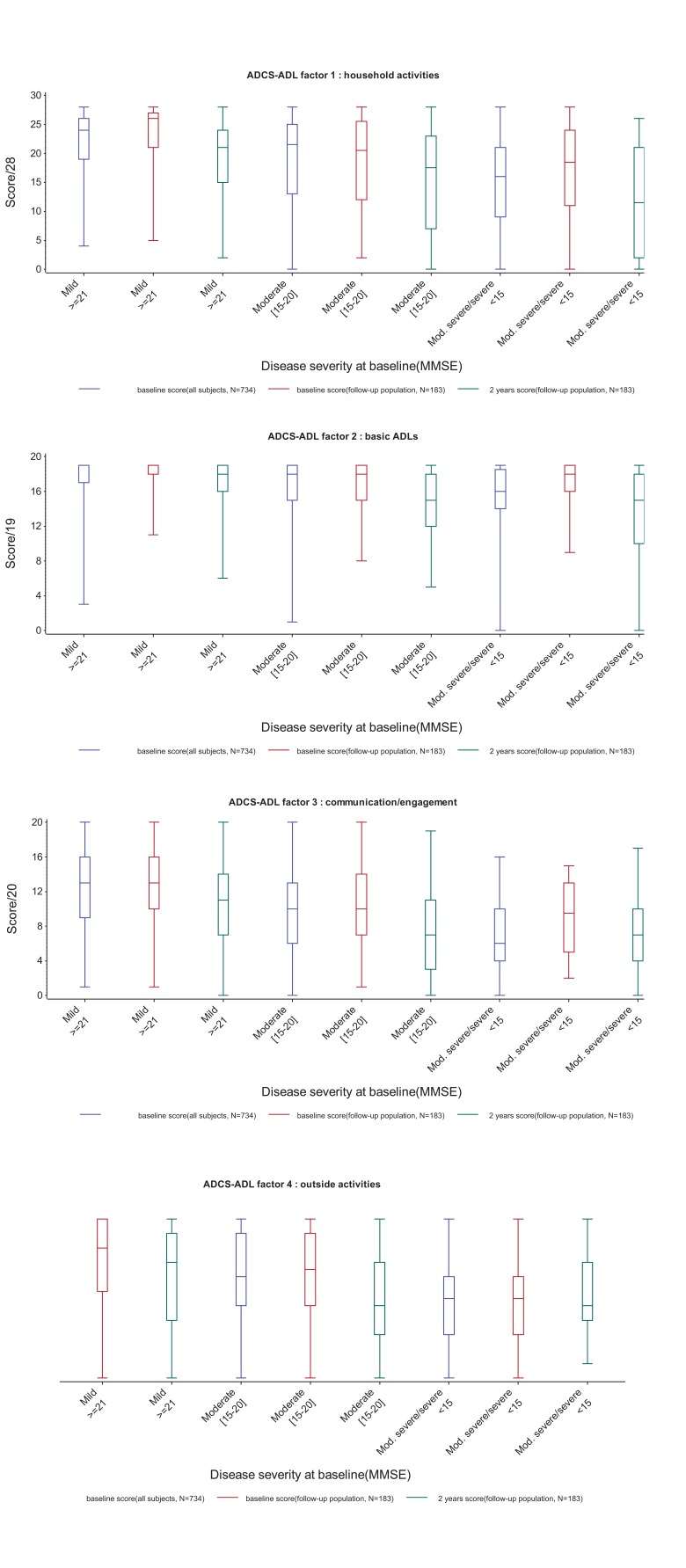
Baseline and 2-year scores for the 4 factors identified in the baseline analysis, according to disease severity at baseline.

Factor loadings are presented in (Table **4**). Factor 1 represented primarily domestic and household activities. Factor 2 included items representing BADLs. Factor 3 included items related to communication and engagement with the environment. Factor 4 included items related to outside activities (travel and shopping). Three items whose loadings fell below the established cut-off >0.30 were assigned to the factor on which they most highly loaded.

(Fig. **2**) shows boxplots of the four factor scores at baseline and 2 years in subjects with mild (MMSE≥21), moderate

(MMSE 15-20) or moderately severe to severe (MMSE<15) AD at baseline. For all four factors, median scores decreased with increasing disease severity at baseline, and there was a decline in median score between baseline and 2 years. However, scores on all of the factors showed a considerable amount of variability at both time points, and factor 2 (basic ADLs) showed evidence of ceiling effects at baseline, in particular in the mild AD group. 

For the analyses using baseline to endpoint change scores, total eigenvalues of the reduced correlation matrix was 7.39, and the average eigenvalue was 0.30. The analyses of loadings that were greater than 0.30 suggested up to an 8 factor solution for the data according to the mean eigenvalue rule, whereas the scree test did not reveal a clear breaking point to determine the number of factors. Therefore, 2, 3, 4, and 5 factor solutions were considered, as these minimized cross-loadings, non-loadings, and factors with less than 3 loaded items. 

The 2-factor solution yielded a basic ADL factor (Items 1-6a and 6b) and an instrumental ADL factor (Items 7-23), and appeared to provide the best balance between cross-loading, non-loadings, and factors having 3 or more item loadings (See Table **5**). Non-loading items were telephone, dishes, staying alone, keeping appointments, and finding personal belongings. The 4 factor solution for change scores was included in Table **6** as a comparison to the 4 factor solution found using the baseline data. The 4 factor solution with the change score suggested 2 factors that account for instrumental ADLs (IADL) and 2 factors that include basic ADL (BADL) loadings. The other instrumental activities were all grouped together, and the BADLs were broken down into two factors. One basic ADL factor included eating, walking, toileting. The other basic ADL factor included bathing, grooming and dressing. This solution included different item loadings than the baseline data analysis and may be less acceptable than the two factor solution, as one factor includes only 2 items relating to shopping (shopping and paying) and only one fewer non-loading item is noted compared to the 2 factor solution.

## DISCUSSION

Functional ability is an important corollary to cognitive ability in Alzheimer’s disease patients. Of several available tools to quantify functional ability, the ADCS-ADL provides a disease-specific assessment of functional ability. Determining the underlying factor structure of the scale may help clinicians, families, and other key stakeholders better understand this multi-faceted concept.

Theory-based separations of the ADCS-ADL typically divide the scale into two principal factors: basic ADLs and Instrumental ADLs. This division is a useful heuristic, particularly when comparing to other trials that may use different measures of BADLs and IADLs. The four factor solution suggested by the present results confirms the presence of a distinct BADL factor that includes eating, walking, toileting, bathing, grooming, and dressing. Additional IADL factors were also suggested. Two of those factors, household activities and communication activities, included similar factors to those found in the LASER-AD population [11]. However, the present analyses suggested an additional factor of outside activities. This difference between the present study and LASER-AD results may be partially driven by the LASER-AD patients being slightly older (81 vs. 79) and having a lower mean MMSE score (14.7 vs. 19.8) [18], suggesting that the LASER-AD factors may be more relevant to more severe patients. Nonetheless, practitioners may use the four categories presented in the present results when communicating with families about expected changes in functioning associated with the AD disease process. In addition, researchers who use this scale or similar scales in future studies may consider treating these as separate factors in analytic plans.

Reasons for the different structures suggested by the present analyses and prior analyses may be related to the different methodologies used to derive the factors. Whereas Livingston *et al.* appeared to use a conceptually-based approach to dictate their analysis plan (e.g., separating out the BADLs a priori and treating factors as independent via a varimax rotation), the present approach instead used the data properties to dictate the analysis plan. Additionally, the differing results may also be due to the population included in the analyses. Livingston and colleagues also note that participants in the LASER-AD study were highly motivated volunteers who may not be representative of the broader patient population [11].

Results of the analyses have two key limitations. While our study population came from a large trial of patients with AD, our results are limited by the fact that we could not include all subjects in the analyses due to missing data. Although a reliance of completers removes the variance associated with imputed scores, some bias is expected based on the type of persons that are able to provide complete data at baseline and endpoint. Further, only a sub-set of all PLASA participants were included, and this subset was on average younger, more recently diagnosed, and had fewer comorbidities. Also, patients excluded from the follow-up analyses were more functionally impaired at baseline. This limits the generalizability of results and may bias the findings toward a profile of factors more relevant to mild or moderate patients rather than the profile of what one might see in severe patients. While this study suffered from a high level of attrition, as is the case in all studies of AD, many subjects were also excluded from our analyses because they did not complete all of the ADCS-ADL items. We chose not to impute missing items for our analyses, given that our aim was to better understand the properties of the ADCS-ADL scale, but future studies may consider optimal imputation techniques that would best reflect anticipated changes in function over time.

When examining the factor structure that was based on change scores, 4- and 2- factor solutions were considered. The 4 factor solution was considered to provide a comparison with the baseline factor structure. Using change scores did not replicate the baseline factor structure. Instead, the BADLs were broken down into two separate factors and IADLs were divided into 1 broad factor and 1 factor including only items related to shopping. The 2 factor solution for the change scores showed a single BADL factor and a single IADL factor. Results using the change scores should be viewed as exploratory, as the change scores do not reflect the traditional method of scoring the items. As such, applying a CFA type of analysis to change scores may create some difficulty around interpretability of the data. This was noted in the high number of factors indicated by the scree plot and high number of non-loadings in the factorial solutions. Also, the number of participants with longitudinal data was limited (n=183), and small sample sizes increase the likelihood of errors in regard to finding correct factor solutions [14]. 

Three items on the ADCS-ADL did not load well in these analyses at baseline: finding personal belongings, writing, and being able to stay alone. These items were assigned to factors based on their highest coefficient values. When considering the baseline data, these assigned loadings make conceptual sense. It was hypothesized that these low values may reflect a higher degree of shared variance with another item on the same factor. For example, performance on the reading and writing items are likely to be highly associated. However, the correlation between these items, was relatively low (r=0.19, results not shown). These items may not be representative of the same conceptualization of function as other items on the scale. Future iterations of the ADCS-ADL scale or other functional scales may consider whether or not to include these items.

## CONCLUSION

The analyses presented in this study provide additional detail about how ADLs may be understood. Function may be explained in terms of BADLs, household activities, communication/engagement, and outside activities. Although these preliminary analyses suggest that BADLs may not be helpful in assessing change over time in milder patients, assessing changes in household activities, communication, and outside activities may be helpful across the disease spectrum. Future longitudinal studies, including clinical trials, should therefore consider additional validation of these factors by looking at change over time of these separate factors to help elucidate the rate at which these different domains decline, particularly for patients in milder stages of dementia. Such information may help family members and physicians develop better care plans and may provide information on interim endpoints of disease process. Additionally, future studies may look at costs associated with different domains to help understand how declines in various domains of BADLs and IADLs may impact costs of care and quality of life for patients and caregivers.

## Figures and Tables

**Fig. (1):**
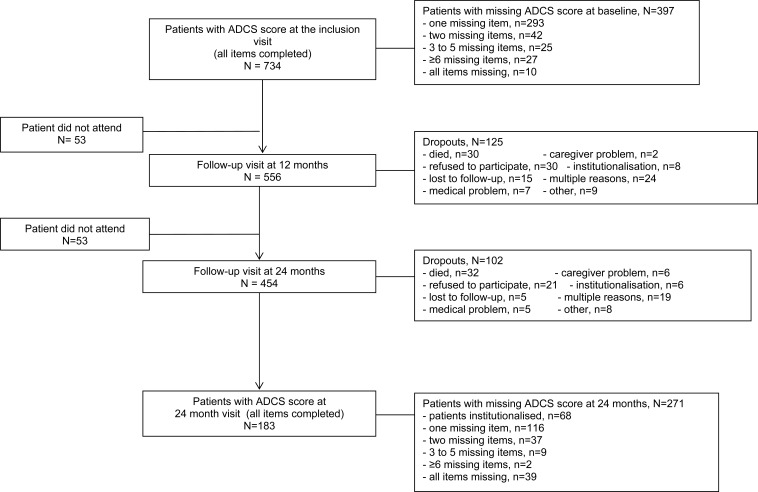
Flow diagram of patient participation in longitudinal study.

**Table 1.** Inter-factor Correlation Matrix at Baseline

	Factor 1	Factor 2	Factor 3	Factor 4
Factor 1	1.00	0.54	0.49	0.54
Factor 2	0.54	1.00	0.34	0.49
Factor 3	0.49	0.34	1.00	0.51
Factor 4	0.54	0.49	0.51	1.00

**Table 2.** aseline Characteristics of the PLASA Cohort Included in Analyses, N=734

Variable	Mean (SD)
Age	79.30 (5.60)
Time since symptom onset (years)	3.55 (2.67)
Time since AD diagnosis (years)	1.32 (1.63)
MMSE	19.83 (3.97)
Total ADCS-ADL	54.46 (14.84)
	N (%)
Female	538 (73.30)
Education Primary school or less Certificate Middle School High school or more	192 (26.23) 294 (40.16) 141 (19.26) 105 (14.34)
Living Situation Alone With spouse With family/Other	248 (33.79) 382 (52.04) 104 (14.17)
Comorbidities (present) Hypercholesterolemia Hypertension Depression	244 (33.38) 347 (47.34) 287 (39.26)
Medications Taking Alzheimer’s treatments (acetylcholinesterase inhibitors and/or memantine) Taking <3 concomitant prescription meds Taking >3 concomitant prescription meds	575 (78.34) 346 (47.14) 388 (52.86)

**Table 3.** Evolution of Disease Severity Over 2 Years in PLASA Subjects Included in the Follow-up Analysis, N=183

Variable	Baseline Score Mean (SD)	2-year Score Mean (SD)
MMSE	20.83 (3.96)	18.20 (5.99)
ADCS-ADL BADL IADL Total score	20.22 (2.79) 38.17 (11.68) 58.39 (13.69)	18.14 (4.53) 31.08 (13.50) 49.21 (16.90)

**Table 4.** Baseline ADCS-ADL Items and Factor Loadings

Item Name (Number)	Factor 1 (eigenvalue =7.12) Household Activities	Factor 2 (eigenvalue =1.34) Basic ADLs	Factor 3 (eigenvalue =0.85) Communication and Engagement	Factor 4 (eigenvalue=0.67) Outside Activities
Eating		0.44		
Walking		0.58		
Toileting		0.66		
Bathing		0.47		
Grooming		0.51		
Dressing – Picking out clothes	0.66			
Dressing - physically getting dressed		0.58		
Telephone	0.38			
Television			0.67	
Conversation			0.47	
Dishes	0.53			
Personal belongings*	0.21			
Drink	0.69			
Cooking snack	0.72			
Litter	0.44			
Travel†				0.46
Shopping				0.77
Paying				0.76
Appointments			0.33	
Alone*				0.22
Current event			0.68	
Reading			0.64	
Writing*			0.26	
Hobbies			0.30	
Appliances	0.67			

**Table 5.** Change Score Factor Loadings: 2-Factor Solution

Item Name (Number)	Factor 1 (eigenvalue =3.51) Instrumental	Factor 2 (eigenvalue =1.27) Basic
Eating		0.45
Walking		0.51
Toileting		0.45
Bathing		0.43
Grooming		0.52
Dressing – Picking out clothes		0.37
Dressing - physically getting dressed		0.70
Telephone*	0.20	
Television	0.49	
Conversation	0.37	
Dishes*	0.28	
Personal belongings*	0.22	
Drink	0.48	
Cooking snack	0.46	
Litter	0.40	
Travel	0.38	
Shopping	0.51	
Paying	0.42	
Appointments*	0.27	
Alone*	0.27	
Current event	0.40	
Reading	0.40	
Writing	0.30	
Hobbies	0.39	
Appliances	0.41	

* Note: Item did not meet criteria of >0.30 loading for any of the factors.

**Table 6.** Change Score Factor Loadings: 4-Factor Solution

Item Name (Number)	Factor 1 (eigenvalue =3.51) Global Instrumental	Factor 2 (eigenvalue =1.27) Basic I	Factor 3 (eigenvalue =0.99) Basic II	Factor 4 (eigenvalue=0.93) Shopping
Eating			0.63	
Walking			0.67	
Toileting			0.42	
Bathing		0.55		
Grooming		0.63		
Dressing – Picking out clothes		0.54		
Dressing - physically getting dressed		0.61		
Telephone*		0.26		
Television	0.44			
Conversation	0.34			
Dishes	0.33			
Personal belongings*	0.20			
Drink	0.55			
Cooking snack	0.38			
Litter	0.49			
Travel†	0.46			
Shopping				0.66
Paying				0.70
Appointments*	0.24			
Alone*	0.30			
Current event	0.32			
Reading	0.38			
Writing	0.31			
Hobbies*	0.29			
Appliances*		0.28		

* Note: Item did not meet criteria of >0.30 loading for any of the factors.

† Note: Item loaded on more than 1 factor so greater loading was retained.
